# Associations of Age and Season at First Calving with First-Lactation Curve Shape in Mediterranean Buffaloes

**DOI:** 10.3390/ani16213522

**Published:** 2026-11-07

**Authors:** Lucia Trapanese, Matteo Santinello, Nadia Piscopo, Roberta Matera, Sissy Purrone, Angela Salzano

**Affiliations:** Department of Veterinary Medicine and Animal Production, University of Naples Federico II, 80137 Naples, Italy; lucia.trapanese2@unina.it (L.T.); matteo.santinello@unina.it (M.S.); roberta.matera@unina.it (R.M.); sissy.purrone@unina.it (S.P.); angela.salzano@unina.it (A.S.)

**Keywords:** age at first calving, breeding, *Bubalus bubalis*, herd management, modelling, milk yield, peak yield, seasonality, wood’s model

## Abstract

This study investigated the effects of age at first calving (AFC), calving season and their interaction on first-lactation curves in Italian Mediterranean buffaloes. Test-day milk yield records collected from 2013 to 2023 were fitted using the Wood model. After excluding atypical curves (22.2%) and data editing, the final dataset comprised 46,289 first lactations and 332,333 test-day records. Generalized and linear mixed models assessed Wood parameters, peak milk yield (Milk_peak_), days to peak (DIM_peak_), and 270-day milk yield (*MY270*). AFC significantly affected *a*, *c* (*p* < 0.01), Milk_peak_ and *MY270* (*p* < 0.001), with *a*, Milk_peak_, and *MY270* increasing consistently with AFC, whereas *c* followed a different pattern. Calving season affected all traits (*p* < 0.001): *a*, Milk_peak_, and *MY270* were lower in summer and autumn, while *b* and DIM_peak_ were higher in summer and *c* in winter. The interaction between AFC class and calving season was significant for all variables (*p* < 0.001, except *b* and *c*, *p* < 0.05; DIM_peak_ *p* < 0.01) except *a* (*p* = 0.460). At AFC ≤ 30 months, summer-calving buffaloes had lower *MY270* and Milk_peak_ but higher DIM_peak_, *b* and *c* than spring-calving buffaloes. Year affected all traits (*p* < 0.001), with values increasing until 2021 before slightly declining. These results indicate that AFC influences the productive level of primiparous animals that exhibit typical lactation curves, while calving season affects both lactation dynamics and production, thus helping farmers optimize first-calving timing.

## Simple Summary

This study examined the association of age at first calving (AFC) and calving season with the characteristics of the first-lactation milk yield curve in Italian Mediterranean buffaloes. Individual lactation curve parameters were estimated by fitting the Wood model to test-day milk yield records from primiparous buffaloes. After excluding curves with atypical 22.2% shapes and cleaning the dataset, 46,289 lactations were retained for the analysis. Buffaloes calving for the first time at more than 42 months produced more milk than those calving at 30 months or younger, both at peak (11.05 vs. 10.27 kg/day, respectively) and over 270 days (2276 vs. 2135 kg, respectively). The advantage of an older age of calving was more pronounced in summer and autumn (0.79 vs. 0.98 kg/day at peak; 159 vs. 165 kg over 270 days respectively for summer and autumn) compared to winter and spring (0.60 vs. 0.76 kg/day; 115–122 kg respectively, for winter and spring). These results showed that, in dairy buffaloes that exhibit typical curves, AFC and calving season are associated with first lactation characteristics and may be useful when planning the timing of first calving.

## 1. Introduction

Italian Mediterranean buffalo (*Bubalus bubalis*) is the predominant dairy buffalo breed in Europe and plays an important economic role in the Italian dairy industry. Buffalo milk is primarily processed into Mozzarella di Bufala Campana cheese [1], whose supply chain has been reported to generate more than €300 million annually with an expanding international market [2]. In this context, reproductive management plays a key role in the economic efficiency of dairy buffalo farming. Among the factors related to reproductive management, age at first calving (AFC) and calving season are particularly relevant, as AFC determines the length of the pre-productive period and the timing at which animals begin generating returns through milk production, while calving season contributes to shaping the seasonal distribution of milk production [3]. Moreover, AFC is associated with genetic turnover, productive longevity and overall farm profitability [4,5].

In Italian Mediterranean buffaloes, AFC has progressively decreased from approximately 42 to 35–36 months [4], mainly through improvements in heifers’ nutritional and reproductive management, with further reductions considered achievable under optimized management conditions [6,7]. However, earlier calving may involve a trade-off between continued growth and milk production, as buffaloes entering their first lactation before reaching adequate somatic and mammary development, may have greater nutrient requirements for growth, potentially limiting milk production. Previous research in Italian Mediterranean buffaloes has shown that the association between AFC and productive and economic performance varies across animals productive life [5]. Although total milk yield during first lactation tends to increase with AFC, this advantage does not persist across subsequent lactations. Indeed, first calving either before or after approximately 35–38 months was associated with less favorable long-term productive and economic outcomes, suggesting that maximizing first-lactation milk yield does not translate into better lifetime performance [5].

These findings highlighted the importance of considering how milk production evolves throughout the first lactation. Although cumulative milk yield provides an overall measure of productivity, it does not distinguish whether differences among AFC classes arise from changes in milk peak yield, the timing of milk peak yield, or the subsequent decline in milk production. Modelling individual lactation curves may therefore provide a more detailed understanding of the associations between AFC and lactation dynamics. Among the models used to describe lactation curves, Wood’s incomplete gamma function has been widely applied across dairy species [8]. This model is characterized by three parameters: parameter *a* represents a scale factor associated with milk production level, parameter *b* describes the increasing component of the curve, and parameter *c* describes the exponentially declining component. Together, these parameters allow the estimation of biologically relevant characteristics, including peak milk yield, time to peak and cumulative milk production.

Besides AFC, calving season may also contribute to differences in lactation dynamics, as buffalo reproductive activity and the environmental conditions experienced during lactation are strongly influenced by seasonality. Buffaloes undergo marked reproductive seasonality and are generally considered short-day breeders. Under italian environmental conditions, fertility increases during periods of decreasing day length, whereas reproductive activity is generally lower during spring and summer [7]. However, this reproductive pattern does not coincide with the seasonal demand for buffalo milk, which is particularly high in spring and summer due to the increased demand for Mozzarella di Bufala Campana. Consequently, out-of-season breeding strategies are often adopted to redistribute calvings and milk production towards these periods [7]. As a result, some buffaloes begin lactation during warmer months, when elevated environmental temperatures may adversely affect milk production. Consistent with this, Catillo et al. [9] reported lower milk yields in summer-calving buffaloes, particularly during early lactation, suggesting that environmental conditions around calving may influence the early trajectory of milk production. Such differences could be reflected in the scale and shape of the lactation curve, particularly Wood parameters *a* and *b*, and potentially in peak milk yield and time to peak. However, their analysis was based on lactation curves from 534 buffaloes and did not directly evaluate associations between calving season and individual Wood parameters. Whether these seasonal differences are also reflected in specific lactation curve parameters and derived characteristics across a larger population therefore remains unclear.

Accordingly, this study evaluated the associations of AFC, calving season and their interaction with Wood model parameters and derived lactation-curve characteristics in primiparous Italian Mediterranean buffaloes. We hypothesized that AFC would primarily affect the production scale of the lactation curve, reflecting differences in somatic and mammary maturity at first calving, whereas calving season would influence both initial milk production and the ascending phase of lactation through seasonal variation in environmental conditions during early lactation.

## 2. Materials and Methods

Ethical approval from an animal welfare and use committee was not required for this study, as data were obtained from routine animal recording practices conducted by the Italian National Association of Buffalo Breeders (ANASB).

### 2.1. Data and Editing

Data consisted of routine AT4 test-day records collected approximately every four weeks between January 2013 and December 2023 within the official Italian Breeders Association (AIA) milk-recording system and according to ICAR standards. Under the AT4 scheme, milk yield is recorded at a single milking event, alternating between morning and evening milkings, using approved milk-recording devices. The corresponding 24-h milk yield is then estimated and expressed as kg/d. AFC was calculated as the interval between birth and first calving dates, expressed in months by dividing the number of days by 30.43. Following the editing criteria adopted in previous studies [3,5,10], buffaloes with AFC values < 23 or >47 months were excluded to limit the influence of potentially erroneous or extremely early records and delayed first calvings potentially associated with management decisions, including seasonal breeding strategies. The AFC was then categorized into eight classes: ≤30, >30–≤32, >32–≤34, >34–≤35, >35–≤37, >37–≤38, >38–≤42 and >42 months, following the procedure described by Calanni Macchio et al. [3]. This categorization has been performed to obtain balanced numbers of observations while maintaining thresholds considered biologically relevant for reproductive management.

Concerning the editing, only records from primiparous buffaloes were retained. To ensure that herd-test-day groups were sufficiently representative and to limit the influence of very small contemporary group size on the analysis, only groups including at least five lactating buffaloes recorded on the same test-day were retained. Moreover, only lactations with at least four test-day records and with the first test-day recorded within 70 days in milk (DIM) were considered eligible for curve fitting. To evaluate the appropriateness of this threshold, a sensitivity analysis was performed using more restrictive criteria of 45 and 60 DIM. Model fitting performance was assessed using the root mean square error (RMSE), while the proportion of lactation curves classified as atypical was also evaluated. The RMSE remained unchanged across thresholds (1.06), whereas the proportion of atypical curves decreased from 22.2% at 70 DIM to 21.6% and 20.0% at 60 and 45 DIM, respectively. Given the limited differences in fitting performance and the substantial reduction in the number of eligible lactations associated with stricter thresholds, the 70-DIM criterion was retained as a compromise between data availability and model fitting performance. The resulting dataset included 62,367 first lactations from 306 herds, comprising 442,733 test-day records (approximately 7 records per lactation).

### 2.2. Lactation-Curve Fitting and Derived Traits

The individual curves were fitted using the Wood incomplete gamma function:

*Y*(*t*) = *a* ⋅ *t^b^* ⋅ *e*^−^*^c^*^⋅^*^t^* t ≥ 0

where Y(*t*) represents the predicted daily milk yield (kg/day) at time *t* expressed as DIM (days) measured from calving (*t* = 0); *a* is the scale parameter associated with starting milk yield level, *b* describes the rate of increase during the ascending phase of lactation and *c* describes the rate of decline after milk peak. Parameter estimation was performed in Python 3.13.7 [11] using the “curve_fit” function of the SciPy package version 1.16.2 [12,13]. No bounds were imposed on the parameters to avoid restricting the optimization to predefined parameter ranges. Accordingly, the default Levenberg–Marquardt algorithm was used. Initial values for the Wood parameters (*a*, *b* and *c*) were derived from Trapanese et al. [14], who fitted the Wood function to milk records from Italian Mediterranean buffaloes. In that study, the resulting mean parameter estimates were adopted as population-specific starting values in the present analysis to facilitate convergence. All remaining optimization settings were kept at their default values, including the convergence tolerance (ftol = 1.49 × 10^−8^) and the maximum number of function evaluations (maxfev = 800). Fitted curves were subsequently classified as either typical or atypical according to the signs of the estimated parameters. Curves with *a* > 0, *b* > 0 and *c* > 0 were classified as typical, as these conditions describe the conventional lactation pattern characterized by an initial increase in milk yield, a positive and finite time to peak production (*t* = *b*/*c*), and a subsequent decline. This classification is based on the mathematical properties of the Wood function. All other parameters combinations were classified as atypical [15] and excluded from subsequent analyses. Out of the 62,367 fitted lactation curves, 13,855 (22.2%) were classified as atypical, leaving 48,512 typical curves. To assess potential selection bias associated with this exclusion, the occurrence of atypical curves was examined across AFC classes, calving seasons and their combinations (Tables S1–S3). Associations were evaluated using chi-square tests of independence and their strength was quantified using Cramér’s V [16]. Although atypical curve occurrence was significantly associated with both AFC class and calving season, the magnitude of these associations was negligible (Cramér’s V = 0.020 and 0.055, respectively). The proportion of atypical curves ranged from 21.0% to 23.8% across AFC classes and from 19.5% in winter to 25.0% in summer (Tables S1–S3). Lactation curve characteristics (LCC) such as peak milk yield (Milk_peak_), time to peak milk yield (DIM_peak_), and cumulative 270-day milk yield (*MY270*) were subsequently calculated from the estimated Wood parameters as follows:

$$\mathrm{DI}M_{\mathrm{peak}}=\frac{b}{c}$$

$$\mathrm{Mil}k_{\mathrm{peak}}=a\cdot {\mathrm{DIM}}_{\mathrm{peak}}^{b}\cdot e^{-c\cdot \mathrm{DI}M_{\mathrm{peak}}}$$

$$\mathrm{MY}270=\int_{0}^{270}a\cdot t^{b}\cdot e^{-\mathrm{ct}}\;\mathrm{dt}$$

Following the exclusion of atypical lactation curves, an additional editing step was performed to limit the influence of extreme values on subsequent statistical analyses. Specifically, lactations with values falling outside the 0.5th–99.5th percentile range for any of the Wood parameters (*a*, *b* and *c*) or LCCs were excluded. This procedure resulted in the removal of another 2223 lactations (4.6% of the 48,512 retained after excluding atypical curves). Thus, the final dataset comprised 46,289 first lactations and 332,333 test-day records.

### 2.3. Statistical Analysis

The effects of AFC and calving season on the Wood curve parameters and calculated variables were investigated using R (version 4.3.3; https://www.r-project.org/, accessed on 20 May 2026) [17]. Since parameters *a*, *b* and *c* were not normally distributed and exhibited right-skewed distributions (Figure S1), generalized linear mixed models were fitted using the R package “glmmTMB” (version 1.1.7). The gamma distribution was selected to account for the positive, right-skewed nature of the parameters, providing a more appropriate representation of their distributional characteristics.

The model can be expressed as:

*y_ijkl_* | *u_l_* ~ *Gamma* (*μ_ijkl_*, *ϕ*)

*log*(*μ_ijkl_*) = *β*_0_ + *AFC_i_* + *Calving season_j_* + *Calving year_k_* + (*AFC* × *Calving Season*)*_ij_*+ *u_l_*

where

$$u_{l}∼N\left(0,\sigma_{\mathrm{Herd}}^{2}\right)$$

Y*_ijkl_* is the Wood model parameter being analyzed (*a*, *b* or *c*), *μ_ijkl_* = *E*(*Y_ijkl_* | *u_l_*) is the conditional expected value of the parameter (*a*, *b*, or *c*) being analyzed; *β*_0_ is the intercept.

*AFC_i_* is the fixed effect of the *ith* AFC class (*i* = from 1 to 8); *Calving season_j_* is the fixed effect of the *jth* calving season (*j* = winter: December–February; spring: March–May; summer: June–August; autumn: September–November); (*AFC* × *Calving season*)*_ij_* is the fixed interaction effect between AFC classes and calving season; *Calving Year_k_* is the fixed effect of the *kth* calving year; *u_l_* is the random effect of the herd, *ϕ* is the dispersion parameter of the Gamma distribution, assuming Var (Y*_ijkl_*/*u_l_*) = *ϕμ^2^_ijkl_*.

Linear mixed models, fitted using the R package “lme4” (version 1.1.32), were used to analyze DIM_peak_, Milk_peak_ and *MY270*. The models can be expressed as follows:

*y_ijkl_ = μ + AFC_i_ + Calving season_j_ + Calving Year_k_ + *(*AFC × Calving Season*)*_ij_ + Herd_l_ + ε_ijkl_*

where *y_ijkl_* is the dependent variable Milk_peak_, DIM_peak_ and MY270; *μ* is the population mean for the evaluated trait; *AFC_i_* is the fixed effect of the *i^th^* AFC class (*i* = from 1 to 8); *Calving season_j_* is the fixed effect of the *j^th^* calving season (*j* = winter: December–February; spring: March–May; summer: June–August; autumn: September–November); (*AFC × Calving season*)*_ij_* is the fixed interaction effect between AFC classes and calving season; *Calving Year_k_* is the fixed effect of the *k^th^* calving year; *Herd_l_* represents the random intercept associated with the *l^th^* herd ∼ *N* (0, σ^2^_Herd_), where σ^2^_Herd_ is the herd variance; and *ε_ijkl_* is the random residual ∼*N* (0, σ^2^_e_), where σ^2^_e_ is the residual variance. The inclusion of herd as a random intercept accounted for between-herd variability and the correlation among observations from animals within the same herd, which may share environmental conditions, feeding practices and management strategies. Residual normality and homoscedasticity were assessed using diagnostic plots (Figure S2). To evaluate the appropriateness of this classification, alternative model specifications treating AFC as a continuous variable with either linear or quadratic effects were also tested (Table S4). Model comparisons based on the Akaike Information Criterion (AIC) and Bayesian Information Criterion (BIC) did not identify a single parameterization that consistently provided the best fit across all lactation curve traits. Therefore, AFC was retained as a categorical variable to facilitate biological interpretation and provide estimates that could be more readily translated into practical reproductive management recommendations. Results are presented as estimated marginal means (EMMs) with their associated standard errors (SE). It is worth highlighting that, in the context of linear mixed models, EMMs correspond to least-squares means (LSM). For the Wood curve parameters (*a*, *b* and *c*), EMMs are reported on the original response scale after back-transformation from the log scale. Tests of fixed effects, herd variance and intraclass correlation coefficients (ICCs) for the Wood parameters and lactation curve characteristics are reported in Tables S5 and S6. Šidák-adjusted pairwise comparisons were conducted, and differences were considered statistically significant at *p* < 0.05.

## 3. Results and Discussion

### 3.1. Wood Fitting and Statistics Description

The Wood model described individual lactation dynamics in primiparous buffaloes, with 77.8% of curves having biologically plausible parameter combinations. The remaining 22.2% were classified as atypical and excluded from subsequent analyses because their parameter estimates did not describe a biologically plausible trajectory. The occurrence of atypical curves is not uncommon in buffaloes and reflects the marked heterogeneity of individual lactation patterns reported in this species. Macciotta et al. [18], for example, found that approximately 30% of individual lactation curves of Italian Mediterranean buffaloes fitted with the Wilmink function lacked the conventional peak-shaped profile. Similarly, Şeahin et al. [19], using the Wood model in Anatolian buffaloes, classified 41.2% of lactations as atypical, whereas a proportion of 9.60% has been reported in Nili Ravi buffaloes [20]. Differences among studies may partly reflect variation in breed, production environment, management and the number and quality of available test-day data. In particular, delayed recording of the first test day relative to calving, incomplete records and disturbances in lactation progression have been associated with an increased occurrence of atypical curves [18,21]. Thus, the proportion observed in the present study appears compatible with the substantial individual variability previously described for buffalo lactations.

Descriptive statistics of Wood parameters and derived traits are reported in Table 1. The model achieved a mean R^2^ of 0.73 ± 0.22, indicating substantial variability in goodness of fit across individual lactations, with few curves showing a limited agreement between observed and predicted milk yields. Such variability was consistent with previous studies showing that mathematical functions generally fit average buffalo lactation patterns more consistently than individual curves. The Wood scaling parameter *a*, which is related to the overall level of milk production at the beginning of the modelled lactation trajectory, averaged 4.79 ± 3.13. The large between-animal variation observed for this parameter is not unexpected, as markedly different estimates have been reported across buffalo populations (from 0.35 in the buffalo population of Bangladesh to 49.23 in the Egyptian buffaloes [22,23]). Direct comparison among studies should be interpreted cautiously because its magnitude is affected by differences in population, parity, production level, recording scheme and model implementation.

Parameter *b*, which is associated with the ascending phase of the lactation curve, averaged 0.37 ± 0.28 and was within the range previously reported for buffaloes [24]. Parameter *c*, describing the rate of decline after peak yield, averaged 0.006 ± 0.003. This estimate was slightly higher than values reported by Anwar et al. [25] and Khan et al. 2023 [20] (0.002 and 0.004, respectively), further illustrating the variability in lactation dynamics among buffalo populations. In the present population, DIM_peak_ was reached at 56.4 ± 25 DIM. This relatively late peak is consistent with the lactation pattern described for primiparous Mediterranean buffaloes, which generally reach peak production later than multiparous animals [26]. The estimated Milk_peak_ averaged 11.40 ± 2.40 kg/day, a value comparable with those previously reported for Mediterranean buffaloes [27] and higher than estimates reported for Bangladesh populations managed in the semi-intensive system fed with natural pasture [23]. Finally, the mean *MY270* averaged 2354 ± 514 kg, which was consistent with production levels described for Mediterranean buffalo herds managed under comparable conditions [28,29]. Overall, these results indicated that the derived LCCs provided biologically interpretable descriptors of lactation performance that can subsequently be used to investigate how factors such as AFC and calving season influence lactation dynamics.

### 3.2. Sources of Variation in a, b and c Parameters and Lactation Curve Characteristics Derived from the Wood Lactation Curve

Table 2 shows the effects of AFC, calving season, their interaction and year on Wood parameters (*a*, *b*, *c*) and LCC. AFC class significantly affected the Wood parameters *a* and *c* (*p* < 0.01), as well as Milk_peak_ and *MY270* (*p* < 0.001), whereas it did not significantly affect the *b* parameter (*p* = 0.277) and DIM_peak_ (*p* = 0.080). Calving season significantly influenced all parameters examined (*p* < 0.001). The interaction between AFC class and calving season was significant for Milk_peak_ and *MY270* (*p* < 0.001), as well as for traits *b* and *c* (*p* < 0.05) and DIM_peak_ (*p* < 0.01). No significant interaction was observed for trait *a* (*p* = 0.460). Calving year significantly affected all traits (*p* < 0.001).

The average lactation curves of primiparous buffaloes by AFC class are shown in Figure S3. Regarding the effect of AFC on Wood parameters and LCC, parameter *a* increased with AFC, from 4.37 ± 0.06 in buffaloes calving for the first time at 30 months or less to 4.71 ± 0.08 in those calving at more than 42 months (Table 3). Since parameter *a* represents the scaling component of the Wood function and is related to milk production during the initial phase of lactation [8], these results suggested that buffaloes calving at a more advanced age tended to start their first lactation at a higher production level. Consistent with the pattern observed for Wood parameter *a*, both Milk_peak_ and *MY270* increased from the AFC class ≤ 30 months to the AFC class > 42 months, rising from 10.27 ± 0.08 to 11.05 ± 0.08 kg/d and from 2136.0 ± 17.1 to 2276.5 ± 18.0 kg, respectively. Zicarelli et al. [6], in a study including 953 buffaloes, observed a positive correlation between AFC and milk production during the first lactation, although the magnitude of this association decreased in subsequent lactations. Similarly, Rautela et al. [30] reported lower productive performance in Murrah buffaloes calving at 30–35 months than in animals calving at older ages, with the highest milk yield observed between 36 and 41 months. Calanni Macchio et al. [3] likewise reported greater milk yield in Italian Mediterranean buffaloes with a more advanced AFC. The higher milk production observed in buffaloes with greater AFC may be associated with more advanced physiological maturity and mammary gland development at first calving. Conversely, buffaloes calving at a younger age may have less developed mammary secretory tissue, potentially limiting milk production during their first lactation [31]. AFC significantly affects parameter *c* (*p* < 0.01), indeed Table 3 shows that *c* is different among AFC classes without a clear trend with age. The lowest values were observed in heifers calving at 30–32 months (0.00604 ± 0.00007), and the highest in those calving at >34–≤35 months (0.00631 ± 0.00008).

### 3.3. Effect of Calving Season on Wood Parameters and Lactation Curve Characteristics

The average lactation curves of primiparous buffaloes by AFC class are shown in Figure S4. The effect of calving season on the parameters derived from the Wood model is presented in Table 4. Calving season significantly influenced all parameters (*p* < 0.001). Wood parameter *a* was lowest in summer (4.25 ± 0.06) and autumn (4.46 ± 0.07), whereas higher values were observed in spring (4.87 ± 0.06) and winter (4.64 ± 0.06). Milk_peak_ and *MY270* followed a similar seasonal pattern of Wood parameter *a*, with significantly (*p* < 0.05) lower values in summer and autumn than in spring and winter. Milk_peak_ was 10.56 ± 0.08 and 10.51 ± 0.08 kg/d in autumn and summer respectively, compared with 10.96 and 10.97 ± 0.08 kg/d in spring and winter. Similarly, *MY270* was lower in summer (2172.6 ± 17.0 kg) and autumn (2191.3 ± 17.5kg) than in spring (2257.1 ± 16.8 kg) and winter (2267.2 ± 16.6 kg). These seasonal differences are consistent with previous literature describing the potential influence of heat stress on buffalo physiology [32]. However, the contribution of heat stress to the observed production patterns could not be directly assessed in the present study.

Calving season also affected Wood parameter *b* (*p* < 0.001), which was lowest in spring (0.351 ± 0.004), intermediate in winter and autumn (0.365 ± 0.004 and 0.369 ± 0.005, respectively) and highest in summer (0.396 ± 0.005). This pattern can indicate differences among seasons in the shape of the ascending phase of the lactation curve. Summer-calving buffaloes reached DIM_peak_ later (59.64 ± 0.51 DIM) than spring-calving buffaloes (54.56 ± 0.48 DIM).

Importantly, the higher *b* value in summer should not be interpreted as evidence of superior lactation performance. Rather, together with the lower *a* value and delayed peak, it described a lactation trajectory starting from a lower production level and increasing more gradually towards maximum yield, followed by a slightly faster post-peak decline (higher *c*). One possible biological explanation is that buffaloes calving under warmer environmental conditions may allocate a greater proportion of energy towards thermoregulation and maintenance, potentially delaying the metabolic and endocrine adaptations required to support maximum mammary secretory activity [33,34]. Nevertheless, because direct indicators of heat load and metabolic status were not evaluated, this mechanism should be regarded as a plausible interpretation rather than a demonstrated causal pathway. Overall, calving season mainly influenced the production scale and early trajectory of the lactation curve, supporting the presence of marked seasonal variation in first-lactation dynamics in Italian Mediterranean buffaloes [35].

### 3.4. Effect of the Interaction of AFC and Calving Season on Wood Parameters and Lactation Curve Characteristics

**Figure 1:**
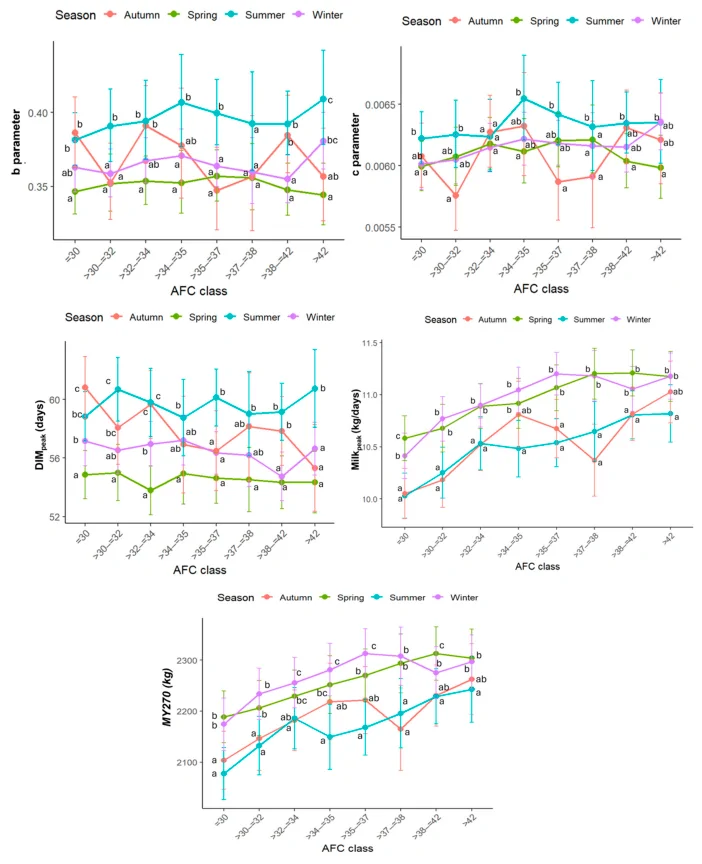
Estimated Marginal Means (EMMs) of Wood parameters (*a*, *b*, *c*) and LCC ^1^ according to the interaction effect between age at first calving (AFC class) and calving season. ^1^ LCC = Lactation curve characteristics were calculated as follows: $\mathrm{DI}M_{\mathrm{peak}}=\frac{b}{c}$, $\mathrm{Mil}k_{\mathrm{peak}}=a\cdot {\mathrm{DIM}}_{\mathrm{peak}}^{b}\cdot e^{-c\cdot \mathrm{DI}M_{\mathrm{peak}}}$ and $\mathrm{MY}270=\int_{0}^{270}a\cdot t^{b}\cdot e^{-\mathrm{ct}}\mathrm{dt}$. ^abc^ letters indicate statistically significant differences among seasons within the same AFC class (*p* < 0.05).

The interaction between AFC class and calving season was significantly associated with parameter *b* and *c* (*p* < 0.05), DIM_peak_ (*p* < 0.01), Milk_peak_, and *MY270* (*p* < 0.001), indicating that the association between AFC and productive performance differed according to the season of first calving. For Milk_peak_ and *MY270*, both AFC and calving season showed clear effects (Figure 1). Within each season, buffaloes calving at >42 months produced more than those calving at ≤30 months, but the difference was not constant. Indeed, for Milk_peak_ the difference was greatest in autumn (+0.98 kg/d), intermediate in summer and winter (+0.79 and +0.77 kg/d) and lowest in spring (+0.59 kg/d), whereas for *MY270* the difference reached 170 and 159 kg in summer and autumn, respectively compared with 125 and 115 kg in winter and spring, respectively. DIM_peak_ was reached earliest in spring (54–55 DIM in all AFC classes) and latest in summer (59–61 DIM in seven of the eight classes). The parameters *b* followed the same pattern (lowest in spring, 0.344–0.357; highest in summer, 0.381–0.409), whereas winter and autumn were intermediate (56.5 and 57.9 DIM; b: 0.354–0.380 and 0.347–0.391). Parameter *c* varied less among seasons (spring 0.00599, autumn 0.00609, winter 0.00619 and summer 0.00634). The results suggested that the summer season accelerates the decline in the lactation curve, whereas no clear pattern is evident across AFC classes. The interpretation of the results is limited to typical lactation curves and cannot be extended to atypical lactation curves.

These interaction patterns complemented the main effects described above. AFC was primarily associated with the production level of the lactation curve, as indicated by differences in parameter *a* and by the generally greater milk production of buffaloes calving for the first time at an older age. In contrast, calving season was associated with differences in the early trajectory of lactation, particularly through parameter *b* and DIM_peak_.

From a biological perspective, the larger AFC-associated differences observed in summer and autumn may indicate that younger primiparous buffaloes are more susceptible to unfavorable seasonal conditions around the onset of lactation. This interpretation is consistent with Gómez-Carpio et al. [4], who reported a particularly marked seasonal effect during early lactation, with the lowest milk production in summer-calving buffaloes. Younger primiparous animals may be especially vulnerable because the energetic demands of first lactation coincide with continued somatic development and mammary maturation. Under challenging environmental conditions, these concurrent requirements could potentially amplify the productive disadvantage associated with an earlier AFC [36]. However, because body development, feeding intake, body condition, metabolic status and individual heat-load indicators were not measured in the present study, this mechanism cannot be directly demonstrated and should be regarded as a plausible explanation for the observed interactions.

### 3.5. Effect of the Calving Year on Wood Parameters and Lactation Curve Characteristics

The year of first calving significantly affects all variables (*p* < 0.001). Table 5 reported that Milk_peak_ and *MY270* increased by about 11% between 2013 and 2021 (from 10.20 ± 0.09 to 11.26 ± 0.08 kg/d and from 2089.6 ± 19.1 to 2349.5 ± 17.1 kg, respectively) and decreased slightly thereafter. This trend is in line with the improvement in average milk yield per lactation reported by the ANASB in the work of Cesarani et al. [37] and with the phenotypic trend of 270-d milk yield described by Gómez-Carpio et al. [10], which increased until 2011, dropped until 2015 and then recovered. The higher production level was accompanied by changes in the shape of the curve, with parameters *a*, *b* and *c* increasing and with the peak being reached later (from 55.93 ± 0.71 DIM in 2013 to 58.88 ± 0.54 DIM in 2020). However, the year effect also includes changes in management, feeding, climate and recording practices, which cannot be separated in the present study.

### 3.6. Management Implications

The present results provided a basis for developing more accurate production scenarios in dairy buffalo simulation models. Because lactation performance varies according to AFC, calving season and year, incorporating these factors into model parameter estimations could allow a better representation of daily milk production under different management and environmental conditions. From a practical perspective, the difference in *MY270* between AFC ≤30 and >42 months suggests that optimizing AFC through adequate heifer growth and development may help achieve an appropriate productive potential at first calving. This result should be interpreted as evidence of a greater first-lactation productive potential rather than as a recommendation to delay first calving. The present study was restricted to primiparous buffaloes and did not include an economic evaluation; therefore, the higher *MY270* observed at older AFC does not account for the additional rearing time and costs associated with delayed first calving, nor for their consequences on subsequent reproductive performance, longevity and lifetime productivity. Calving season is also relevant in relation to the timing of mating. Out-of-season breeding strategies are widely adopted, as they aim to reduce summer–autumn calvings in favour of winter–spring calvings, which are expected to be followed by more profitable lactations [7]. Our results are consistent with this rationale: in primiparous buffaloes, calvings in winter and spring were associated with higher Milk_peak_ and *MY270* than those in summer and autumn. However, the potential productive advantage should be weighed against the reduced fertility of out-of-season mating.

## 4. Conclusions

The present study demonstrated that the Wood model can provide biologically meaningful descriptors of lactation dynamics in a large population of primiparous Mediterranean buffaloes. AFC significantly affected the *a* parameter, Milk_peak_, *MY270*, and the *c* parameter, but had no significant effect on the *b* parameter orDIM_peak_. Overall, the findings suggested that buffaloes calving at a more advanced age tend to exhibit greater milk production than those calving at a younger age. Calving season significantly affected all variables describing the shape of the lactation curve. Buffaloes calving for the first time in summer exhibited the lowest values for the *a* parameter, Milk_peak_, and *MY270* compared with those calving in spring or winter. Summer calving was also associated with a higher DIM_peak_. The interaction between AFC and calving season further revealed that younger buffaloes calving in the summer had the lowest Milk_peak_ and *MY270* values, whereas higher values were observed in older buffaloes calving in spring and autumn. In contrast, the *b* and *c* parameters and DIM_peak_ appeared to be primarily influenced by calving season, showing relatively consistent patterns across AFC classes. These findings highlighted the importance of integrating reproductive management decisions with seasonal factors to optimize the productive performance of primiparous Italian Mediterranean buffaloes. However, some limitations should be considered when interpreting these results. First, the analyses were based on parameters and traits derived from the Wood function and therefore depend on the assumptions and accuracy of the fitted model. As a future direction, the performance of alternative lactation curve models could be evaluated to identify the model that best describes the lactation curve of Italian Mediterranean buffaloes. Second, 22.2% of the fitted curves were classified as atypical (*a*, *b* or *c* not positive) and excluded. Although the occurrence of atypical curves was only weakly associated with AFC and calving season, the occurrence of atypical curves could have slightly affected the representativeness of summer lactations in the final dataset. However, given the negligible strength of this association, it is unlikely to have substantially biased the seasonal estimates. The exclusion of atypical lactation curves does not allow us to assess whether the patterns observed in this study remain consistent across typical and atypical lactations. Moreover, the study is observational and referred to first lactation only; the possible effects of AFC on later productivity, fertility and longevity and the costs of delaying first calving were not evaluated in this study. Future studies should include additional information on body condition score, body weight, feed intake, environmental indexes and relevant physiological indicators to better investigate the potential factors underlying the observed associations between AFC, calving season and lactation performance in Italian Mediterranean buffaloes.

## Figures and Tables

**Table 1:** Descriptive statistics of Wood model parameters (*a*, *b* and *c*), coefficient of determination (R^2^), and lactation curve characteristics (LCC ^1^).

Parameters	N.	Mean ± SD	Median	IQR ^2^	Minimum	Maximum
*a*	46,289	4.79 ± 3.13	4.37	2.37–6.74	0.00659	15.2
*b*	46,289	0.37 ± 0.28	0.31	0.18–0.48	0.005	2.05
*c*	46,289	0.006 ± 0.003	0.006	0.0041–0.0078	0.0006	0.02
R^2^	46,289	0.73 ± 0.22	0.80	0.61–0.91	0.03	0.99
Milk_peak_ (kg/day)	46,289	11.4 ± 2.40	11.3	9.69–12.99	5.63	18.91
DIM_peak_ (days)	46,289	56.4 ± 25.0	54.8	40.36–70.53	1.95	189
MY270 (kg)	46,289	2354 ± 514	2320	1992–2686	1065	3988

^1^ LCC = Lactation curve characteristics were calculated as follows: $\mathrm{DI}M_{\mathrm{peak}}=\frac{b}{c}$, $\mathrm{Mil}k_{\mathrm{peak}}=a\cdot {\mathrm{DIM}}_{\mathrm{peak}}^{b}\cdot e^{-c\cdot \mathrm{DI}M_{\mathrm{peak}}}$ and $\mathrm{MY}270=\;\int_{0}^{270}a\cdot t^{b}\cdot e^{-\mathrm{ct}}\;\mathrm{dt}.$ ^2^ IQR = Interquartile range.

**Table 2:** Effects of age at first calving (AFC), calving season, their interaction, and year on Wood parameters (*a*, *b*, *c*) and lactation curve characteristics (LCC ^1^). Wood parameters were analyzed using Gamma generalized linear mixed models, while peak milk yield (Milk_peak_), days in milk at peak (DIM_peak_), and 270-day milk yield (*MY270*) were analyzed using linear mixed models. Statistical significance was assessed using Wald χ^2^ tests for generalized linear mixed models and F-tests with Satterthwaite’s approximation for Linear mixed models.

**Effects**	**a**			**b**			**c**		
	***p*** **-Value**	**Wald χ^2^**	**RMSE**	***p*** **-Value**	**Wald χ^2^**	**RMSE**	***p*** **-Value**	**Wald χ^2^**	**RMSE**
AFC class (months)	<0.01	23.19	3.03	0.277	8.67	0.28	<0.01	21.827	0.003
Calving season	<0.001	45.29		<0.001	108.37		<0.001	29.162	
AFC class × Calving season	0.460	20.98		<0.05	33.78		<0.05	34.320	
Year	<0.001	42.51		<0.001	75.27		<0.001	132.651	
**Effects**	**Milk_peak_ (kg/day)**			**DIM_peak_ (days)**			**MY270 (kg)**		
	***p*** **-Value**	**F-Value**	**RMSE**	***p*** **-Value**	**F-Value**	**RMSE**	***p*** **-Value**	**F-Value**	**RMSE**
AFC class (months)	<0.001	75.43	2.00	0.080	1.82	24.08	<0.001	51.92	422.60
Calving season	<0.001	111.99		<0.001	63.31		<0.001	93.40	
AFC class × Calving season	<0.001	3.08		<0.01	2.01		<0.001	3.05	
Year	<0.001	177.76		<0.001	5.88		<0.001	171.22	

^1^ LCC = Lactation curve characteristics were calculated as follows: $\mathrm{DI}M_{\mathrm{peak}}=\frac{b}{c}$, $\mathrm{Mil}k_{\mathrm{peak}}=a\cdot {\mathrm{DIM}}_{\mathrm{peak}}^{b}\cdot e^{-c\cdot \mathrm{DI}M_{\mathrm{peak}}}$ and $\mathrm{MY}270=\;\int_{0}^{270}a\cdot t^{b}\cdot e^{-\mathrm{ct}}\;\mathrm{dt}$.

**Table 3:** Estimated marginal means (EMMs), standard error (SE) and 95% confidence intervals (CI 95%, Šidák adjusted) of Wood model parameters and LCC ^1^ according to the effect of age at first calving class (AFC ^2^).

Parameters		AFC ^2^ (Months)							
		≤30	30–32	32–34	34–35	35–37	37–38	38–42	>42
N		8042	5626	7154	4208	7975	2883	6252	4149
*a*	EMM	4.37 ^a^	4.44 ^ab^	4.50 ^ab^	4.49 ^ab^	4.61 ^b^	4.63 ^ab^	4.65 ^b^	4.71 ^b^
	SE	0.06	0.07	0.07	0.08	0.07	0.09	0.07	0.08
	CI-95%	4.21–4.54	4.26–4.63	4.32–4.68	4.28–4.71	4.43–4.80	4.39–4.88	4.47–4.84	4.49–4.93
*b*	EMM	0.369	0.363	0.376	0.376	0.366	0.366	0.369	0.372
	SE	0.005	0.005	0.005	0.006	0.005	0.007	0.005	0.006
	CI-95%	0.356–0.383	0.349–0.378	0.362–0.391	0.359–0.394	0.352–0.381	0.348–0.385	0.356–0.384	0.355–0.389
*c*	EMM	0.00608 ^a^	0.00604 ^a^	0.00622 ^ab^	0.00631 ^b^	0.00617 ^ab^	0.00616 ^ab^	0.00622 ^ab^	0.00623 ^ab^
	SE	0.00006	0.00007	0.00007	0.00008	0.00007	0.00008	0.00007	0.00007
	CI-95%	0.00592–0.00626	0.00586–0.00622	0.00604–0.00640	0.00610–0.00652	0.00599–0.00635	0.00594–0.00639	0.00604–0.00640	0.00603–0.00644
DIM_peak_ (days)	EMM	57.91	57.57	57.55	56.96	56.90	56.97	56.51	56.76
	SE	0.52	0.56	0.54	0.62	0.55	0.67	0.53	0.61
	CI-95%	56.50–59.33	56.06–59.09	56.07–59.02	55.26–58.65	55.41–58.39	55.15–58.78	55.06–57.97	55.10–58.42
Milk_peak_ (kg/d)	EMM	10.27 ^a^	10.47 ^b^	10.71 ^c^	10.81 ^cd^	10.87 ^de^	10.85 ^cde^	10.97 ^ef^	11.05 ^f^
	SE	0.08	0.08	0.08	0.08	0.08	0.09	0.08	0.08
	CI-95%	10.05–10.48	10.25–10.69	10.49–10.93	10.58–11.04	10.65–11.09	10.62–11.08	10.75–11.19	10.82–11.28
MY270 (kg)	EMM	2136.0 ^a^	2179.6 ^b^	2213.4 ^c^	2225.3 ^cd^	2243.2 ^de^	2240.5 ^cdef^	2262.0 ^ef^	2276.5 ^f^
	SE	17.1	17.5	17.3	18.1	17.4	18.6	17.2	18.0
	CI-95%	2089.3–2182.6	2132.0–2227.2	2166.1–2260.6	2175.9–2274.7	2195.9–2290.6	2189.7–2291.2	2214.9–2309.0	2227.4–2325.6

^1^ LCC = Lactation curve characteristics were calculated as follows: $\mathrm{DI}M_{\mathrm{peak}}=\frac{b}{c}$, $\mathrm{Mil}k_{\mathrm{peak}}=a\cdot {\mathrm{DIM}}_{\mathrm{peak}}^{b}\cdot e^{-c\cdot \mathrm{DI}M_{\mathrm{peak}}}$ and $\mathrm{MY}270=\;\int_{0}^{270}a\cdot t^{b}\cdot e^{-\mathrm{ct}}\;\mathrm{dt}$. ^2^ AFC (months): first: ≤30 mo; second: >30–≤32 months; third: >32–≤34 months; fourth: >34–≤35 months; fifth: >35–≤37 months; sixth: >37–≤38 months; seventh: >38–≤42 months; and eighth: >42 months. ^abcdef^ Means with different superscript letters within the same row differ significantly (*p* < 0.05).

**Table 4:** Estimated marginal means (EMMs), standard error (SE) and 95% confidence intervals (CI 95%, Šidák adjusted) of Wood model parameters and LCC ^1^ according to the effect of season of calving.

Parameters		Calving Season			
		Autumn	Spring	Summer	Winter
N.		5303	13,191	8645	19,150
*a*	EMM	4.46 ^b^	4.87 ^d^	4.25 ^a^	4.64 ^c^
	SE	0.07	0.06	0.06	0.06
	CI-95%	4.29–4.63	4.72–5.02	4.11–4.40	4.51–4.79
*b*	EMM	0.369 ^b^	0.351 ^a^	0.396 ^c^	0.365 ^b^
	SE	0.005	0.004	0.005	0.004
	CI-95%	0.355–0.383	0.341–0.362	0.383–0.409	0.354–0.375
*c*	EMM	0.00610 ^a^	0.00610 ^a^	0.00634 ^b^	0.00617 ^a^
	SE	0.00007	0.00006	0.00006	0.00006
	CI-95%	0.00593–0.00627	0.00596–0.00625	0.00618–0.00650	0.00603–0.00631
DIM_peak_ (days)	EMM	57.90 ^c^	54.56 ^a^	59.64 ^d^	56.47 ^b^
	SE	0.56	0.48	0.51	0.47
	CI-95%	56.50–59.30	53.36–55.76	58.36–60.91	55.31–57.64
Milk_peak_ (kg/d)	EMM	10.56 ^a^	10.96 ^b^	10.51 ^a^	10.97 ^b^
	SE	0.08	0.08	0.08	0.08
	CI-95%	10.36–10.76	10.77–11.16	10.32–10.71	10.78–11.16
MY270 (kg)	EMM	2191.3 ^a^	2257.1 ^b^	2172.6 ^a^	2267.2 ^b^
	SE	17.5	16.8	17.0	16.6
	CI-95%	2147.7–2234.9	2215.3–2298.9	2130.2–2215.0	2225.8–2308.7

^1^ LCC = Lactation curve characteristics were calculated as follows: $\mathrm{DI}M_{\mathrm{peak}}=\frac{b}{c}$, $\mathrm{Mil}k_{\mathrm{peak}}=a\cdot {\mathrm{DIM}}_{\mathrm{peak}}^{b}\cdot e^{-c\cdot \mathrm{DI}M_{\mathrm{peak}}}$ and $\mathrm{MY}270=\;\int_{0}^{270}a\cdot t^{b}\cdot e^{-\mathrm{ct}}\;\mathrm{dt}$. ^abcd^ Means with different superscript letters within the same row differ significantly (*p* < 0.05).

**Table 5:** Estimated marginal means (EMMs), standard error (SE) and 95% confidence intervals (CI 95%, Šidák adjusted) of Wood model parameters and LCC ^1^ according to the effect of calving year.

Parameters		Calving Year										
		2013	2014	2015	2016	2017	2018	2019	2020	2021	2022	2023
N.		2026	3122	3059	3412	2361	3179	4584	5277	6144	7441	5684
*a*	EMM	4.40 ^a^	4.45 ^a^	4.42 ^a^	4.44 ^a^	4.68 ^ab^	4.55 ^ab^	4.58 ^ab^	4.64 ^ab^	4.78 ^b^	4.61 ^ab^	4.51 ^a^
	SE	0.09	0.08	0.08	0.08	0.09	0.08	0.07	0.07	0.07	0.06	0.07
	CI-95%	4.15–4.67	4.23–4.67	4.20–4.64	4.23–4.66	4.43–4.94	4.33–4.79	4.38–4.79	4.45–4.84	4.59–4.97	4.43–4.79	4.32–4.70
*b*	EMM	0.362 ^abc^	0.353 ^a^	0.361 ^abc^	0.365 ^abc^	0.356 ^ab^	0.368 ^abc^	0.377 ^bc^	0.380 ^cd^	0.373 ^abc^	0.379 ^c^	0.397 ^d^
	SE	0.007	0.006	0.006	0.006	0.007	0.006	0.006	0.005	0.005	0.005	0.006
	CI-95%	0.342–0.383	0.336–0.370	0.344–0.378	0.348–0.382	0.338–0.375	0.351–0.386	0.362–0.394	0.365–0.395	0.359–0.387	0.365–0.393	0.382–0.413
*c*	EMM	0.00623 ^ab^	0.00608 ^a^	0.00604 ^a^	0.00608 ^a^	0.00603 ^a^	0.00609 ^a^	0.00614 ^a^	0.00610 ^a^	0.00623 ^ab^	0.00635 ^b^	0.00662 ^c^
	SE	0.00009	0.00007	0.00007	0.00007	0.00008	0.00007	0.00007	0.00006	0.00006	0.00006	0.00007
	CI-95%	0.00598–0.00648	0.00588–0.00630	0.00583–0.00625	0.00588–0.00629	0.00581–0.00626	0.00588–0.00631	0.00595–0.00633	0.00592–0.00629	0.00605–0.00641	0.00617–0.00653	0.00643–0.00682
DIM_peak_ (days)	EMM	55.93 ^ab^	55.72 ^a^	57.08 ^abcd^	57.26 ^abcd^	56.41 ^ab^	57.87 ^bcd^	58.53 ^cd^	58.88 ^d^	56.90 ^ab^	56.89 ^ab^	57.08 ^abc^
	SE	0.71	0.62	0.62	0.61	0.67	0.62	0.56	0.54	0.52	0.51	0.54
	CI-95%	53.93–57.92	53.96–57.48	55.32–58.84	55.54–58.97	54.51–58.31	56.12–59.62	56.94–60.12	57.35–60.42	55.42–58.38	55.46–58.32	55.97–58.60
Milk_peak_ (kg/d)	EMM	10.20 ^a^	10.08 ^a^	10.17 ^a^	10.44 ^b^	10.61 ^bc^	10.74 ^c^	11.01 ^d^	11.20 ^e^	11.36 ^f^	11.18 ^e^	11.26 ^ef^
	SE	0.09	0.08	0.08	0.08	0.09	0.08	0.08	0.08	0.08	0.08	0.08
	CI-95%	9.95–10.45	9.85–10.32	9.93–10.41	10.20–10.67	10.36–10.85	10.51–10.98	10.78–11.23	10.98–11.43	11.13–11.58	10.96–11.40	11.04–11.49
MY270 (kg)	EMM	2089.6 ^a^	2077.3 ^a^	2108.6 ^a^	2165.6 ^b^	2201.4 ^bc^	2230.8 ^c^	2289.7 ^d^	2337.0 ^e^	2349.5 ^e^	2298.6 ^d^	2294.4 ^d^
	SE	19.1	18.1	18.1	18.0	18.7	18.1	17.6	17.3	17.1	17.0	17.2
	CI-95%	2035.6–2143.7	2026.0–2128.7	2057.3–2160.0	2114.7–2216.5	2148.5–2254.3	2179.6–2282.1	2240.0–2339.3	2288.0–2386.0	2301.0–2398.0	2250.6–2346.6	2245.7–2343.2

^1^ LCC = Lactation curve characteristics were calculated as follows: $\mathrm{DI}M_{\mathrm{peak}}=\frac{b}{c}$, $\mathrm{Mil}k_{\mathrm{peak}}=a\cdot {\mathrm{DIM}}_{\mathrm{peak}}^{b}\cdot e^{-c\cdot \mathrm{DI}M_{\mathrm{peak}}}$ and $\mathrm{MY}270=\;\int_{0}^{270}a\cdot t^{b}\cdot e^{-\mathrm{ct}}\;\mathrm{dt}$. ^abcdef^ Means with different superscript letters within the same row differ significantly (*p* < 0.05).

## Data Availability

Data will be made available on reasonable request.

## Supplementary Materials

The following supporting information can be downloaded at https://www.mdpi.com/article/10.3390/ani16213522/s1, Table S1: Number of total and atypical curves according to calving season. Table S2: Number of total and atypical curves according to age at first calving class (AFC class). Table S3: Number of total and atypical curves according to age at first calving class (AFC class) and calving season. Table S4: Comparison of model fit statistics for alternative parameterizations of age at first calving (AFC) as a categorical, linear, or quadratic effect on Wood parameters (*a*, *b* and *c*) and lactation curve characteristics (LCCs). Table S5: Degrees of freedom (DF num) or fixed effects in generalized mixed model for wood parameters (*a*, *b* and *c*). Models included AFC class, calving season, their interaction and year of calving with herd as a random effect. Herd variance and intraclass correlation coefficient (ICC) describe between-herd variability. Table S6: Numerator and denominator degrees of freedom (DF num and DF den) or fixed effects in linear mixed model for LCC ^1^. Models included AFC class, calving season, their interaction and year of calving with herd as a random effect. Herd variance and intraclass correlation coefficient (ICC) describe between-herd variability. Figure S1: Distribution of the Wood parameters, LCC ^1^ after the trimming of the after quantile-based trimming. Values below the 0.5th percentile and above the 99.5th percentile of each variable were excluded (n = 46,289). Figure S2: Distribution of the Wood parameters, LCC ^1^ after the trimming of the after quantile-based trimming. Values below the 0.5th percentile and above the 99.5th percentile of each variable were excluded (n = 46,289). Figure S3: Average lactation curves (milk yield, kg/day) of primiparous Italian Mediterranean buffaloes according to calving season. Figure S4: Average lactation curves (milk yield, kg/day) of primiparous Italian Mediterranean buffaloes according to age at first calving classes (AFC) ^1^.

## Abbreviations

The following abbreviations are used in this manuscript:

AFC	Age at First Calving
DIM	Days in Milk
EMM	Estimated Marginal Means
LCC	Lactation Curve Characteristics
LSM	Least Square Means
MY270	Milk Yield at 270 days
