# Supplementary material for: Associations of Age and Season at First Calving with First-Lactation Curve Shape in Mediterranean Buffaloes
Source: Animals (Basel). 2026 Nov 7;16(21):3522. doi: 10.3390/ani16213522 (PMC13762169; doi:10.3390/ani16213522)

**Associations of age and season at first calving with first-lactation curve shape in Mediterranean buffaloes**

**Lucia Trapanese [1], Matteo Santinello[1], Nadia Piscopo[1]*, Roberta Matera[1], Sissy Purrone[1], Angela Salzano[1]**

[1]Department of Veterinary Medicine and Animal Production, University of Naples Federico II, 80137 Naples, Italy

* Correspondence: nadia.piscopo@unina.it

## Supplementary Materials

**Supplementary Table S1**. Number of total and atypical curves according to calving season.

| Calving season | N. of total curves | N. of atypical curves | Atypical curves (%) |
|---|---|---|---|
| **Summer** | 12,230 | 3,058 | 25.0 |
| **Spring** | 18,061 | 4,283 | 23.7 |
| **Autumn** | 7,266 | 1,683 | 23.2 |
| **Winter** | 24,810 | 4,831 | 19.5 |

**Supplementary Table S2.** Number of total and atypical curves according to age at first calving class (AFC class).

| AFC Class | N. of total curves | N. of atypical curves | Atypical curves (%) |
|---|---|---|---|
| ≤30 | 10,905 | 2,454 | 22.5 |
| 30-32 | 7,525 | 1,623 | 21.6 |
| 32-34 | 9,583 | 2,099 | 21.9 |
| 34-35 | 5,586 | 1,174 | 21.0 |
| 35–37 | 10,690 | 2,343 | 21.9 |
| 37–38 | 3,818 | 800 | 21.0 |
| 38–42 | 8,558 | 2,005 | 23.4 |
| >42 | 5,702 | 1,357 | 23.8 |

**Supplementary Table S3**. Number of total and atypical curves according to age at first calving class (AFC class) and calving season.

| Season | AFC class | N. of total curves | N. of atypical curves | Atypical curves (%) |
|---|---|---|---|---|
| Autumn | ≤30 | 1,537 | 347 | 22.6 |
| | 30-32 | 1,002 | 232 | 23.2 |
| | 32-34 | 1,193 | 266 | 22.3 |
| | 34-35 | 517 | 128 | 24.8 |
| | 35–37 | 803 | 185 | 23.0 |
| | 37–38 | 402 | 95 | 23.6 |
| | 38–42 | 1,138 | 260 | 22.8 |
| | >42 | 674 | 170 | 25.2 |
| Spring | ≤30 | 3,357 | 772 | 23.0 |
| | 30-32 | 1,913 | 421 | 22.0 |
| | 32-34 | 3,060 | 721 | 23.6 |
| | 34-35 | 1,533 | 362 | 23.6 |
| | 35–37 | 2,760 | 681 | 24.7 |
| | 37–38 | 1,373 | 319 | 23.2 |
| | 38–42 | 2,442 | 614 | 25.1 |
| | >42 | 1,623 | 393 | 24.2 |
| Summer | ≤30 | 3,131 | 738 | 23.6 |
| | 30-32 | 1,442 | 368 | 25.5 |
| | 32-34 | 1,201 | 303 | 25.2 |
| | 34-35 | 881 | 198 | 22.5 |
| | 35–37 | 1,981 | 532 | 26.9 |
| | 37–38 | 715 | 168 | 23.5 |
| | 38–42 | 1,984 | 510 | 25.7 |
| | >42 | 895 | 241 | 26.9 |
| Winter | ≤30 | 2,880 | 597 | 20.7 |
| | 30-32 | 3,168 | 602 | 19.0 |
| | 32-34 | 4,129 | 809 | 19.6 |
| | 34-35 | 2,655 | 486 | 18.3 |
| | 35–37 | 5,146 | 945 | 18.4 |
| | 37–38 | 1,328 | 218 | 16.4 |
| | 38–42 | 2,994 | 621 | 20.7 |
| | >42 | 2,510 | 553 | 22.0 |

**Supplementary Table S4**. Comparison of model fit statistics for alternative parameterizations of age at first calving (AFC) as a categorical, linear, or quadratic effect on Wood parameters (*a*, *b*, and *c*) and lactation curve characteristics (LCCs).

| Parameters | Models | N. | AIC | BIC | logLik |
|---|---|---|---|---|---|
| *a* | AFC categorical | 46289 | 230304.7 | 230610.7 | −115117.4 |
| | AFC linear | 46289 | 230273.7 | 230369.9 | −115125.9 |
| | AFC quadratic | 46289 | 230279.7 | 230410.9 | −115124.9 |
| *b* | AFC categorical | 46289 | −7838.740 | −7532.747 | 3954.37 |
| | AFC linear | 46289 | −7852.626 | −7756.457 | 3937.313 |
| | AFC quadratic | 46289 | −7846.559 | −7715.419 | 3938.28 |
| *c* | AFC categorical | 46289 | −400754.1 | −400448.1 | 200412 |
| | AFC linear | 46289 | −401141.8 | −401045.7 | 200581.9 |
| | AFC quadratic | 46289 | −401037.2 | −400906.1 | 200533.6 |
| $DIM_{peak}$(days) | AFC categorical | 46289 | 426876.9 | 427182.9 | −213403.4 |
| | AFC linear | 46289 | 426918.1 | 427014.3 | −213448.1 |
| | AFC quadratic | 46289 | 426955.2 | 427086.3 | −213462.6 |
| $Milk_{peak}$ (kg/d) | AFC categorical | 46289 | 197182.7 | 197488.7 | −98556.35 |
| | AFC linear | 46289 | 197199.5 | 197295.7 | −98588.74 |
| | AFC quadratic | 46289 | 197168.7 | 197299.9 | −98569.36 |
| *MY270(kg)* | AFC categorical | 46289 | 692732.9 | 693038.9 | −346331.5 |
| | AFC linear | 46289 | 692990.0 | 693086.2 | −346484.0 |
| | AFC quadratic | 46289 | 692938.4 | 693069.6 | −346454.2 |

[1]LCC = Lactation curve characteristics were calculated as follows: $DIM_{peak} = \frac{b}{c}$, $Milk_{peak} = a \cdot DIM_{peak}^{b} \cdot e^{-c \cdot DIM_{peak}}$ and $MY270 = \int_{0}^{270} a \cdot t^{b} \cdot e^{-ct}\, dt$

**Supplementary Table S5.** Degrees of freedom (DF num) or fixed effects in generalized mixed model for wood parameters (*a, b* and *c*). Models included AFC class, calving season, their interaction and year of calving with herd as a random effect. Herd variance and intraclass correlation coefficient (ICC) describe between-herd variability.

| Parameters | Factor | DF num | Var (herd) | ICC |
|---|---|---|---|---|
| *a* | AFCClass | 7 | 0.024 | 0.049 |
| | Season | 3 | | |
| | Year of calving | 10 | | |
| | AFC Class x Season | 21 | | |
| *b* | AFCClass | 7 | 0.023 | 0.051 |
| | Season | 3 | | |
| | Year | 10 | | |

| | | | | |
|---|---|---|---|---|
| | AFC Class x Season | 21 | | |
| *c* | AFCClass | 7 | 0.023 | 0.010 |
| | Season | 3 | | |
| | Year of calving | 10 | | |
| | AFC Class x Season | 21 | | |

**Supplementary Table S6**. Numerator and denominator degrees of freedom (DF num and DF den) or fixed effects in linear mixed model for LCC[1]. Models included AFC class, calving season, their interaction and year of calving with herd as a random effect. Herd variance and intraclass correlation coefficient (ICC) describe between-herd variability.

| LCC[1] | Factor | DF num | DF Den | Var (herd) | ICC |
|---|---|---|---|---|---|
| $Milk_{peak}$ (kg/ day) | AFCClass | 7 | 46060 | 1.61 | 0.07 |
| | Season | 3 | 46118 | | |
| | Year of calving | 10 | 46136 | | |
| | AFC Class x Season | 21 | 46011 | | |
| $DIM_{peak}$ (days) | AFCClass | 7 | 46117 | 45.32 | 0.28 |
| | Season | 3 | 46205 | | |
| | Year of calving | 10 | 46013 | | |
| | AFC Class x Season | 21 | 46120 | | |
| *MY270* (kg) | AFCClass | 7 | 46056 | 76,319 | 0.30 |
| | Season | 3 | 46112 | | |
| | Year of calving | 10 | 46130 | | |
| | AFC Class x Season | 21 | 46009 | | |

[1]LCC = Lactation curve characteristics were calculated as follows: $DIM_{peak} = \frac{b}{c}$, $Milk_{peak} = a \cdot DIM_{peak}^{b} \cdot e^{-c \cdot DIM_{peak}}$ and $MY270 = \int_0^{270} a \cdot t^b \cdot e^{-ct}\, dt$

**Supplementary Figure S1.** Distribution of the Wood parameters, LCC[1] after the trimming of the after quantile-based trimming. Values below the 0.5th percentile and above the 99.5th percentile of each variable were excluded (n=46,289).

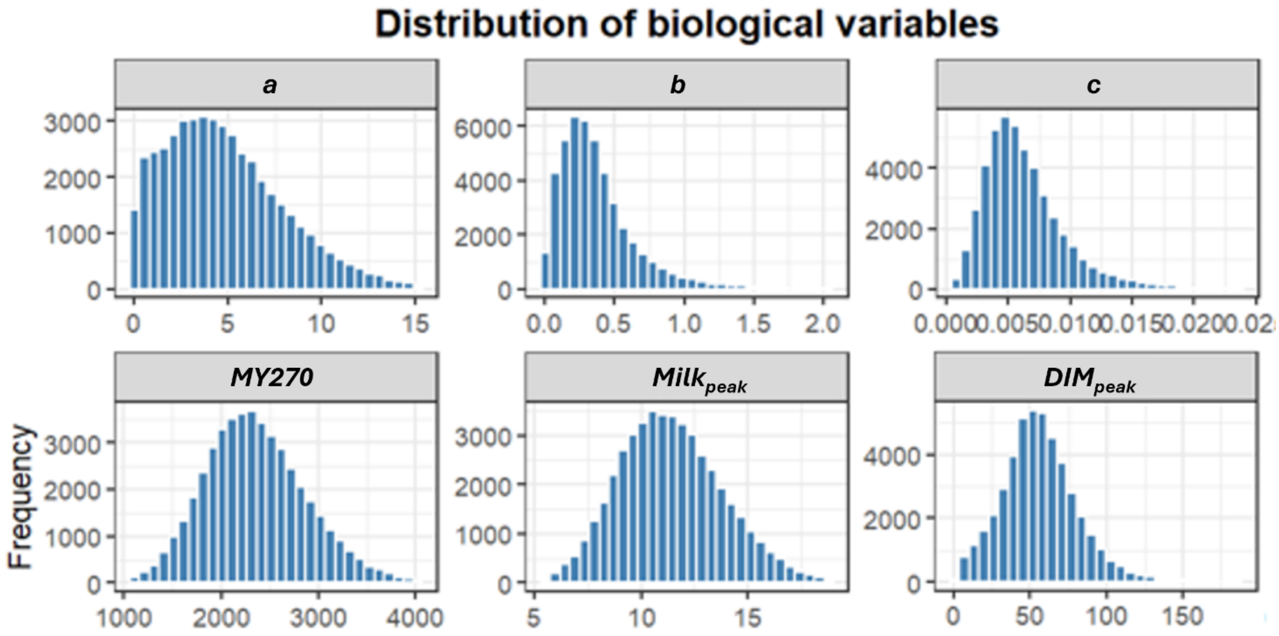


[1]LCC = Lactation curve characteristics were calculated as follows: $DIM_{peak} = \frac{b}{c}$, $Milk_{peak} = a \cdot DIM_{peak}^{b} \cdot e^{-c \cdot DIM_{peak}}$ and $MY270 = \int_0^{270} a \cdot t^b \cdot e^{-ct}\, dt$

**Supplementary Figure S2**. Diagnostic plot of the Wood parameters and LCC[1].

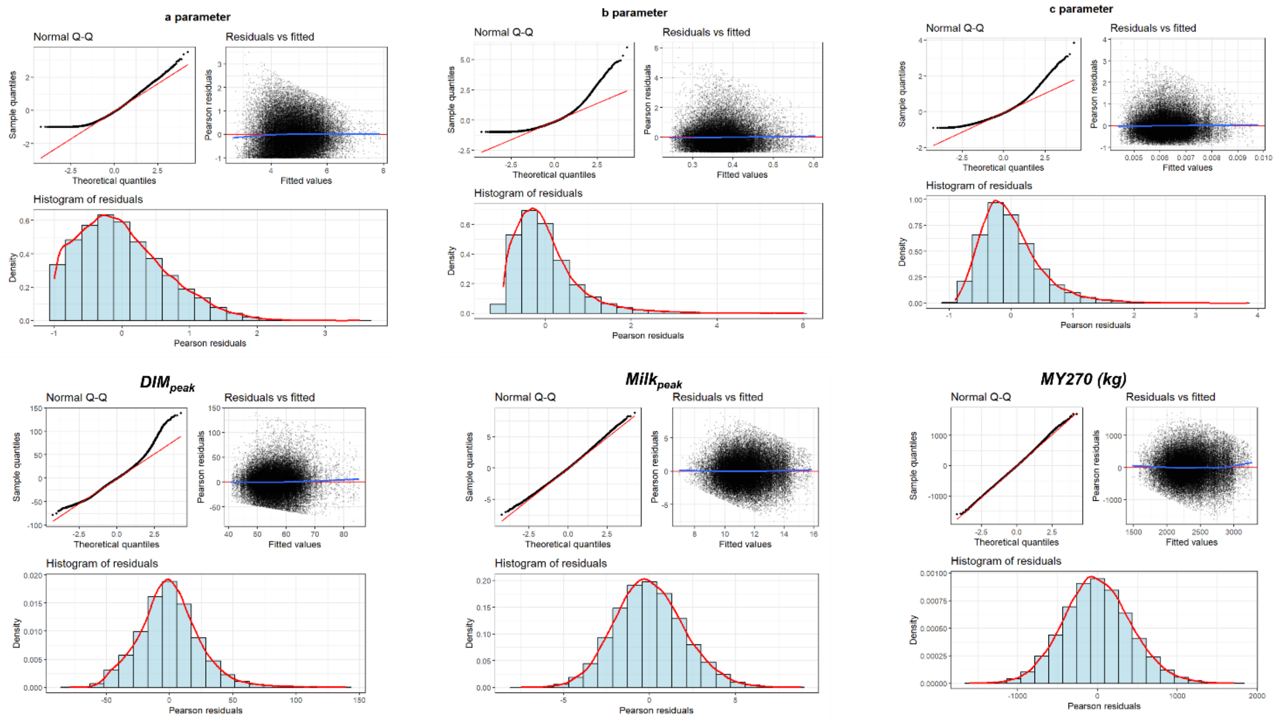


[1]LCC = Lactation curve characteristics were calculated as follows: $DIM_{peak} = \frac{b}{c}$, $Milk_{peak} = a \cdot DIM_{peak}^{b} \cdot e^{-c \cdot DIM_{peak}}$ and $MY270 = \int_{0}^{270} a \cdot t^{b} \cdot e^{-ct}\, dt$

**Supplementary Figure S3**. Average lactation curves (milk yield, kg/day) of primiparous Italian Mediterranean buffaloes according to age at first calving classes (AFC)[1].

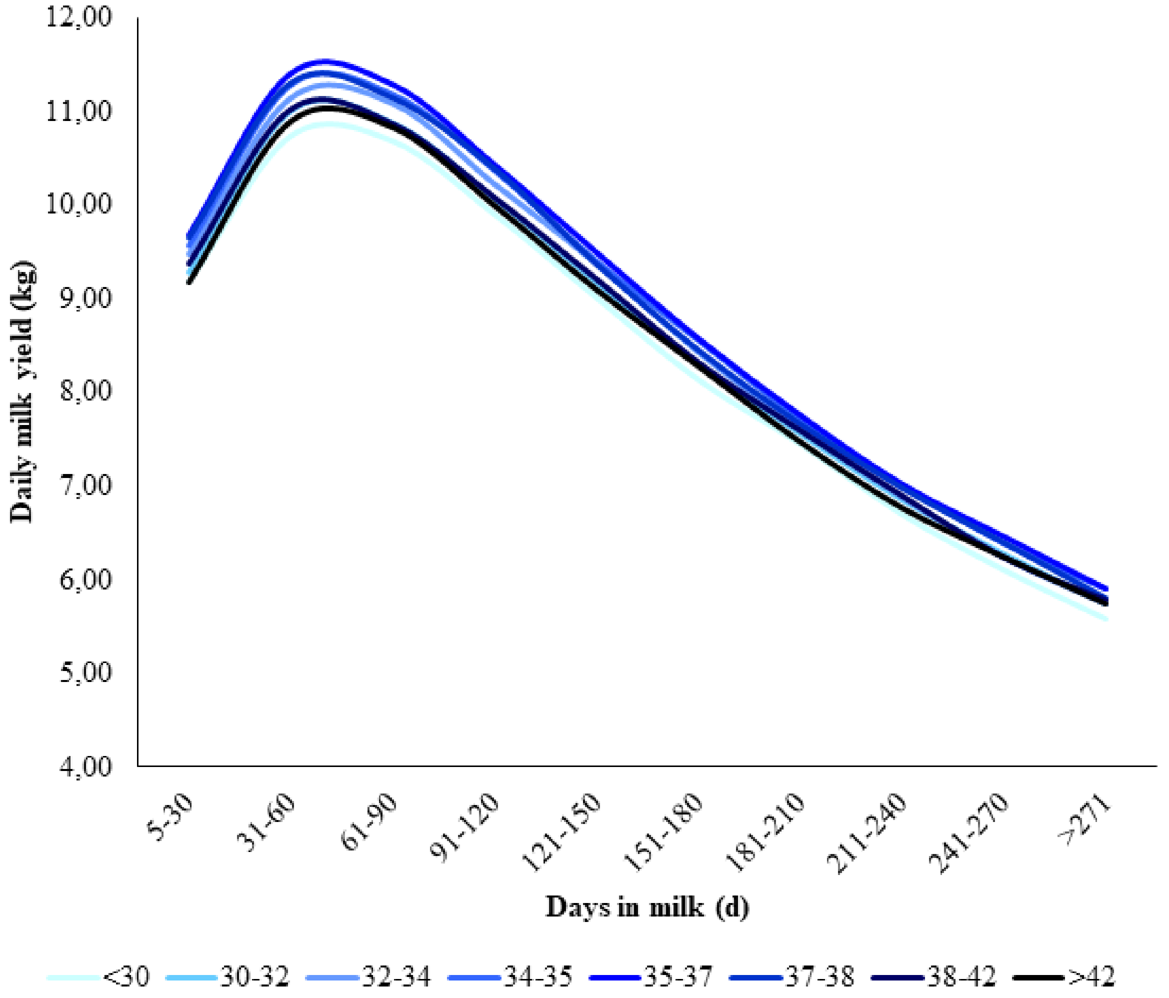


[1]AFC (months): first: ≤30 months; second: 30–32 months; third: 32–34 months; fourth: 34–35 months; fifth: 35–37 months; sixth: 37–38 months; seventh: 38–42 months; and eighth: >42 months.

**Supplementary Figure S4**. Average lactation curves (milk yield, kg/day) of primiparous Italian Mediterranean buffaloes according to the season of calving.

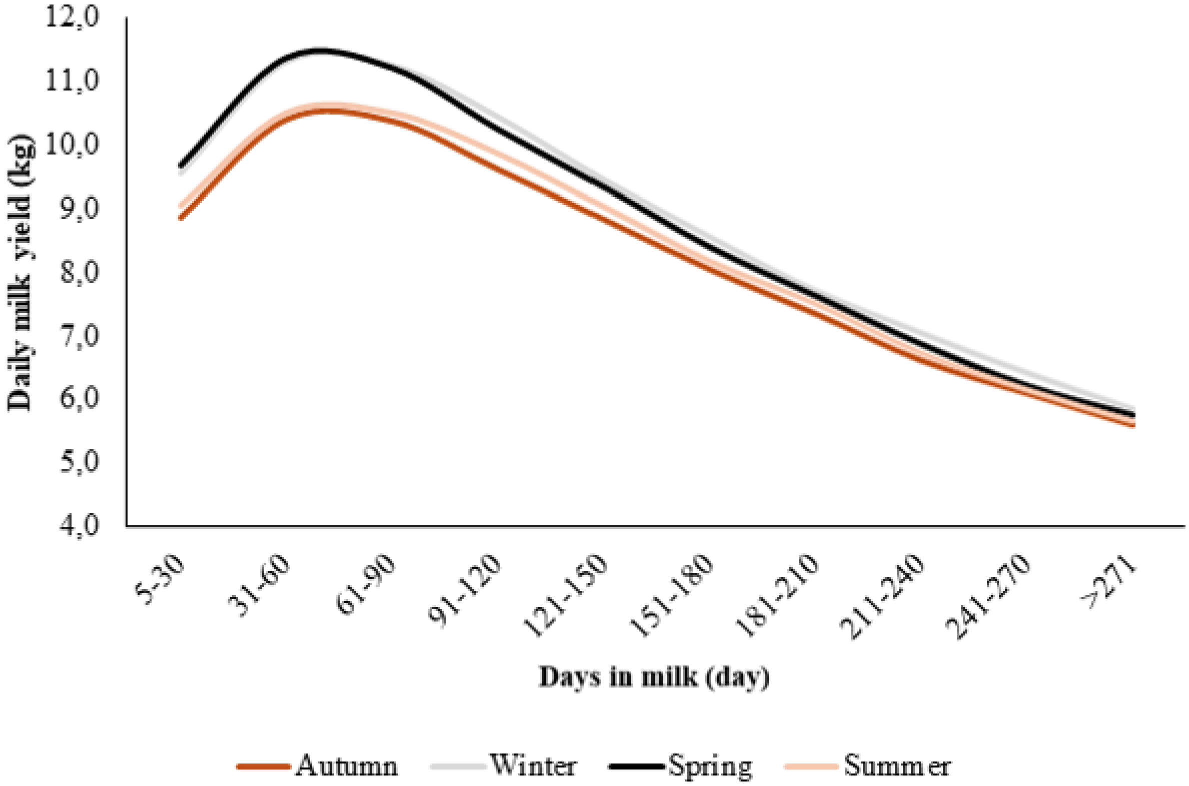

Supplement: Supplementary file 1 [file animals-16-03522-s001.zip › animals-4580501-supplementary.pdf]
